# The NLRP3 and NLRP1 inflammasomes are activated in Alzheimer’s disease

**DOI:** 10.1186/s13024-016-0088-1

**Published:** 2016-03-03

**Authors:** Marina Saresella, Francesca La Rosa, Federica Piancone, Martina Zoppis, Ivana Marventano, Elena Calabrese, Veronica Rainone, Raffaello Nemni, Roberta Mancuso, Mario Clerici

**Affiliations:** Don C. Gnocchi Foundation, IRCCS, Piazza Morandi, 3, 20121 Milan, Italy; Departments of Biomedical and Clinical Sciences “Luigi Sacco”, University of Milano, 20100 Milan, Italy; Departments of Physiopathology and Transplants, University of Milano, 20100 Milan, Italy

**Keywords:** Alzheimer’s disease, Neuroinflammation, Inflammasome, Beta amyloid, Mild cognitive impairment

## Abstract

**Background:**

Interleukin-1 beta (IL-1β) and its key regulator, the inflammasome, are suspected to play a role in the neuroinflammation observed in Alzheimer’s disease (AD); no conclusive data are nevertheless available in AD patients.

**Results:**

mRNA for inflammasome components (NLRP1, NLRP3, PYCARD, caspase 1, 5 and 8) and downstream effectors (IL-1β, IL-18) was up-regulated in severe and MILD AD. Monocytes co-expressing NLRP3 with caspase 1 or caspase 8 were significantly increased in severe AD alone, whereas those co-expressing NLRP1 and NLRP3 with PYCARD were augmented in both severe and MILD AD. Activation of the NLRP1 and NLRP3 inflammasomes in AD was confirmed by confocal microscopy proteins co-localization and by the significantly higher amounts of the pro-inflammatory cytokines IL-1β and IL-18 being produced by monocytes. In MCI, the expression of NLRP3, but not the one of PYCARD or caspase 1 was increased, indicating that functional inflammasomes are not assembled in these individuals: this was confirmed by lack of co-localization and of proinflammatory cytokines production.

**Conclusions:**

The activation of at least two different inflammasome complexes explains AD-associated neuroinflammation. Strategies targeting inflammasome activation could be useful in the therapy of AD.

## Background

Alzheimer’s disease (AD) is a devastating neurodegenerative condition characterized by neuronal cell death and progressive dementia. It is widely accepted that the extracellular accumulation of amyloid-β (Aβ) in senile plaques and the formation of neurofibrillary tangles (NFT) inside neurons as a result of the abnormal phosphorylation of the microtubules-associated tau protein are the main event in the pathogenesis of AD, but the cellular events leading to plaque-induced neuronal dysfunction are less clear [1]. Inflammatory mediators play an essential role in the neuroinflammation observed in AD. In particular interleukin IL-1β is increased in this condition, and the activation of its key regulator, the inflammasome, is suspected to be involved in the pathogenesis of the disease.

The inflammasomes are multiprotein complexes mainly expressed in myeloid cells that are required for the activation of caspase 1 protease and the downstream secretion of two of its substrates, the proinflammatory cytokines IL-1β and IL-18 [2]. Inflammasome-dependent innate immune responses are initiated by Nod-like receptors (NLRs), cytoplasmic pattern recognition receptors that detect invading pathogens. NLRs are activated by bacterial, fungal, or viral molecules that contain pathogen-associated molecular patterns (PAMPs) or by non-microbial danger signals (DAMPs) released by damaged cells [3, 4]. NLR activation leads to their oligomerization to form multiprotein inflammasome complexes that serve as platforms for the recruitment, cleavage, and activation of inflammatory caspases. Four inflammasome complexes (NLRP1, NLRP3, IPAF, and AIM2) have been identified. These complexes contain either a specific NLR family protein or AIM2, as well as the PYCARD and/or Cardinal adaptor proteins and pro-caspases-1, 5 and 8 [5, 6]. NLRP3 is the best-characterized inflammasome; its formation requires multiple steps. In a priming step, transcriptionally active signalling receptors induce the NF-kB-dependent induction of NLRP3 itself as well as that of the caspase 1 substrates of the pro-IL-1β family [7, 8]. The NLRP3 is, at this stage, in a signalling incompetent conformation; this is modified upon a second signal, that will induce the assembly of a multimolecular complex with PYCARD and caspase 1. Multiple signals, which are potentially provided in combination, thus trigger the formation of an active inflammasome, which, in turn, will stimulate the cleavage and the release of bioactive cytokines including IL-1β and IL-18 [8–18]. Although these cytokines have a beneficial role in promoting inflammation and eliminating infectious pathogens, mutations that result in constitutive inflammasome activation and overproduction of IL-1β and IL-18 have been linked to inflammatory and autoimmune disorders [19–21].

The NLRP3 inflammasome complex is suspected to play a role in AD, as its activation in the microglia by Aβ triggers neuroinflammation [14, 22, 23]. Notably, NLRP3 inflammasome deficiency favours the differentiation of microglia cells to an M2 (anti-inflammatory) phenotype and results in a decreased deposition of amyloid-β in the APP/PSI model of AD; these results reinforce the suggestion that the NLRP3 inflammasome is involved in the pathogenesis of the disease [24]. No conclusive data are nevertheless available in patients. To better investigate the involvement of the inflammasome in AD we performed in-depth analyses of the expression of genes and proteins that are part of the inflammasome complex in individuals with a diagnosis of either AD or mild cognitive impairment (MCI). Results indicated that two different inflammasomes, NLRP3 and NLRP1, are activated in AD but not in individuals with a diangnosis of mild cognitive impairment (MCI).

## Results

### Up-regulation of inflammasome genes in LPS-primed and Aβ_42_-stimulated-monocytes of AD patients

**Fig. 1:**
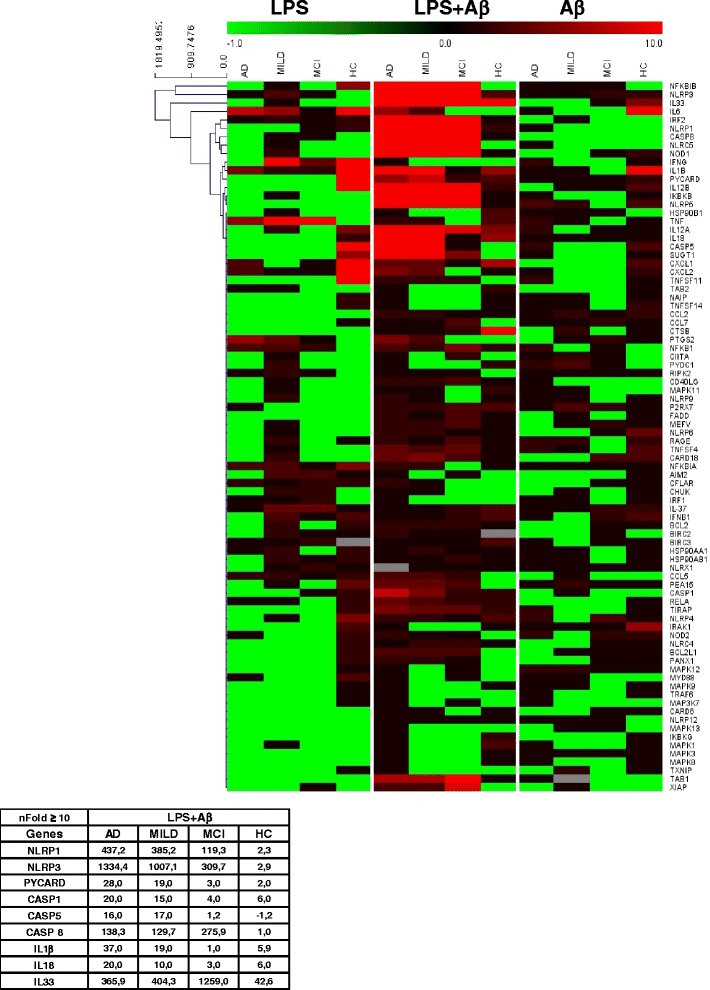
Messenger RNA expression levels of genes within the inflammasome pathway. Expression of 84 genes involved in the inflammasome pathway assessed by real-time quantitative RT-PCR-array in monocytes of individuals with a diagnosis of either severe Alzheimer’s disease (AD), moderate Alzheimer’s disease (MILD) or Mild Cognitive Impairment (MCI) and of age- and sex-matched Healthy Controls (HC). Heat maps of Log_2_ Fold changes are presented. Results obtained upon stimulating cells with LPS or Aβ_42_ alone, or upon LPS-priming followed by Aβ_42_ stimulation are presented. Fold-changes >10 in the expression of LPS + Aβ_42_ stimulated genes of importance are summarized in the Table

mRNA expression of 84 genes involved in the assembly, the activation, and the down-stream signalling of inflammosomes was quantified by qPCR in all patients and controls. Data are expressed as the fold change (nFold) between the results obtained in unstimulated cells and those obtained upon stimulations of cells with either LPS, Aβ42 or LPS and Aβ42. In this initial set of experiments, pools of cells from donors with the same clinical diagnosis (MCI; MILD AD; severe AD) as well as from HC were created. Data obtained in LPS-primed and Aβ_42−_stimulated-monocytes, showed the presence of a significant up-regulation involving genes, that codify for the proteins that form the inflammasome in MILD and severe AD, as well as in MCI. In cells of MCI individuals, though, not all the genes that are necessary for the assembly of a fully functional inflammasome complex were up-regulated. To summarize: 1) the expression of NLRP1, NLRP3 and caspase 8 was greatly increased in all groups of patients (nFold >10) compared to HC, with no detectable differences being observed between individuals with a diagnosis of AD or MCI; and 2) PYCARD, caspase 1 and caspase 5 expression was increased in AD (nFold ≥10) but not in MCI individuals. Only minor differences were seen between patients and controls in the expression of these genes in monocytes-stimulated with either LPS or Aβ_42_ alone; both stimuli induced IL-1β and IL-6 up-regulation in HC (nFold >10) (Fig. 1).

**Fig. 2:**
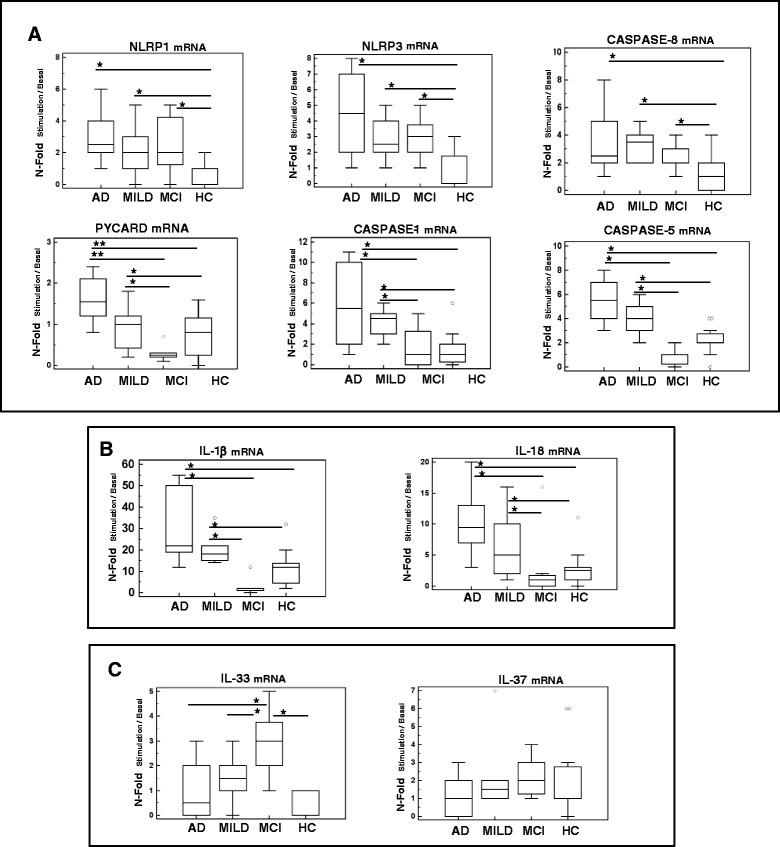
mRNA expression by Real-Time PCR. Single Real-Time PCR results obtained in LPS and Aβ_42_ -stimulated monocytes of individuals with a diagnosis of either severe Alzheimer’s disease (AD), moderate Alzheimer’s disease (MILD) or Mild Cognitive Impairment (MCI) and of age- and sex-matched Healthy Controls (HC). **a** NLRP1, NLRP3, caspase 8, PYCARD, caspase 1 and caspase 5 are shown in panel **a**; IL-1β and IL-18 in panel **b** and IL-33 and IL-37 in panel **c**. The results are shown as fold-change expression from the un-stimulated samples. Gene expression was calculated relative to GAPDH housekeeping gene. Summary results are shown in the bar graphs. The boxes stretch from the 25 to the 75 percentile; the line across the boxes indicates the median values; the lines stretching from the boxes indicate extreme values. Outside values are displayed as separate points. Statistical significance is shown *(*p* <0.05), **(*p* <0.01)

RT-PCR performed on each individual specimen was used next to verify the expression of NLRP1, NLRP3, PYCARD, caspase 1, caspase 5 and caspase 8 in LPS and Aβ_42−_stimulated monocytes of patients and HC. Results of mRNA expression confirmed that: 1) NLRP1, NLRP3 and caspase 8 are significantly increased in AD and MCI individuals compared to HC (*p* <0.05), with the highest values observed in severe AD, and 2) PYCARD, caspase 1 and caspase 5 are significantly increased in AD compared to MCI and HC (*p* <0.05) (Fig. 2a).

### Up-regulation of inflammasome-related cytokines in LPS-primed and Aβ_42−_stimulated- monocytes of AD patients

Stimulation of monocytes with either LPS or Aβ_42_- alone resulted in the moderate increase of IL-1β and IL-6 expression; notably LPS or Aβ_42_- alone were not sufficient to trigger inflammasome assembly, as no differences were detect in these conditions either in NLRP3 or IL-18 mRNA (Fig. 1). Once cells were activated with LPS and Aβ_42_, nevertheless, both IL-1β (nFold >10) and IL-18 (nFold ≥10) mRNA was significantly increased in severe and MILD AD compared to MCI and HC (Fig. 1). Results obtained by single-PCR confirmed that in these experimental conditions IL-1β and IL-18 mRNA (*p* <0.05 vs. all other groups) is significantly increased in severe and MILD AD, with the highest values in severe AD (Fig. 2b).

IL-33 and IL-37, two relatively novel cytokines that are members of the IL-1 family, were analyzed as well in the study. We found that IL-33 gene expression was greatly increased in MCI (nFold >10) compared to AD and HC; no significant differences, on the other hand, were observed in IL-37 gene expression among groups analyzed (Fig. 1). Single RT-PCR confirmed these results (Fig. 2c).

### NLRP3 protein is significantly augmented in LPS-primed and Aβ_42−_stimulated- monocytes of AD and MCI patients

**Fig. 3:**
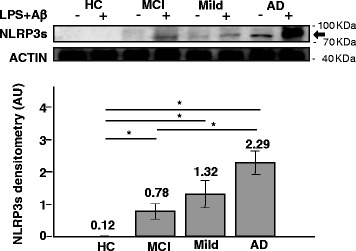
NLRP3s expression in Monocyte by Western blot: NLRP3s protein expression assessed by western blotting in monocyte of individuals with a diagnosis of either severe Alzheimer’s disease (AD), moderate Alzheimer’s disease (MILD) or Mild Cognitive Impairment (MCI) and of age- and sex-matched Healthy Controls (HC). The same protein concentration of whole-cells lysates was loaded into the gel, as confirmed by actin. Representative results obtained in un-stimulated or in LPS-primed and Aβ_42_-stimulated monocytes are presented in the *upper panel*. Quantitative evaluation (arbitrary unit, AU) of NLRP3s expression obtained comparing band density (normalized to actin) in un-stimulated or in LPS-primed Aβ42-stimulated monocytes is shown in the *lower panel*. (*N* = 5, mean ± s.e.m., *T* test, *p <0.05). (*N* = 5/group, mean ± S.E.M and statistical significance are shown. *(*p* <0.05)

The expression of NLRP3 protein, the best-characterized protein within the inflammasome complex, was investigated next by Western Blot analyses in LPS-primed and Aβ42-stimulated-monocytes of all patients and controls. Different NLRP3 isoforms are known: the long form (118 KDa) is prevalent in cell lines (THP1 or Jurkat) while the short one (NLRP3s 75 KDa) is seen in primary immune cells [25]. Results of analyses performed on whole-cell lysates confirmed that NLRP3s expression is significantly increased in severe (mean ± S.E.M.: 2.29 ± 0.35 AU), and MILD AD (1.32 ± 0.42 AU) compared to healthy controls (0.1 ± 0.06 AU)(*p* = 0.023 vs. severe AD; *p* = 0.0362 vs. MILD AD). NLRP3s expression was also increased when MCI individuals (0.78 ± 0.24 AU) were compared to HC, even if this difference did not reach statistical significance (*p* = 0.081) (Fig. 3).

### LPS-primed and Aβ_42−_stimulated monocytes that express inflammasome proteins are significantly augmented in AD

**Fig. 4:**
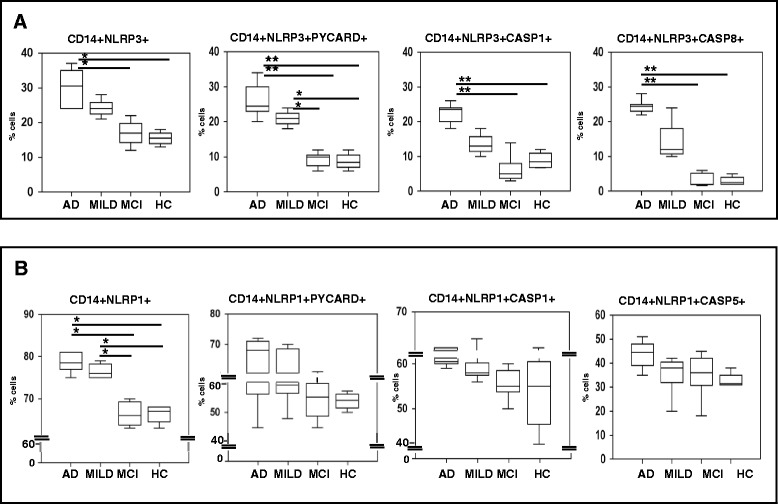
FACS analyses: co-expression of inflammasome proteins in CD14+ cells. Co-expression of inflammasome proteins in LPS-primed and Aβ_42_-stimulated CD14+ of individuals with a diagnosis of either severe Alzheimer’s disease (AD), moderate Alzheimer’s disease (MILD) or Mild Cognitive Impairment (MCI) and of age- and sex-matched Healthy Controls (HC). Two-hundred-thousand cells were acquired and gated on CD14 expression and side scatter properties. CD14+ cells co-expressing NLRP3-associated inflammasome components are shown in panel **a**; CD14+ cells co-expressing NLRP1-associated inflammasome components are shown in panel **b**. Summary results are shown in the bar graphs. The boxes stretch from the 25° to the 75°percentile; the line across the boxes indicates the median values; the lines stretching from the boxes indicate extreme values. Statistical significance is shown *(*p* <0.05), **(*p* <0.01)

Peripheral CD14+ monocytes that express NLRP3 or co-express NLRP3 and PYCARD, NLRP3 and caspase 1, or NLRP3 and caspase 8 were augmented in LPS-primed and Aβ_42−_stimulated-cells of both groups of AD patients compared to MCI individuals and HC. The Fluorochrome Inhibitor of Caspases (FLICA) kit was used to analyze the presence in cells of active caspases; in this method, once inside the cell, the FLICA inhibitor probe binds covalently to active caspases (p20) alone. Staining of active caspase 1 and caspase 8 inhibitors was performed using the green fluorescent probe FAM-YVAD-FMK and FAM-LETD-FMK respectively. Results showed that 1) CD14+/NLRP3+, CD14+/NLRP3+/caspase1+, and CD14+/NLRP3+/Caspase8+ immune cells were significantly increased in severe AD patients alone compared to MCI and HC (*p* <0.05) and 2) CD14+/NLRP3+/PYCARD +, cells were augmented both in severe and MILD AD compared to MCI and HC (MCI and HC vs. moderate AD *p* <0.05; vs. severe AD *p* <0.01)(Fig. 4a).

LPS-primed and Aβ_42_-stimulated NLRP1-expressing CD14 cells were significantly increased as well in both groups of AD patients compared to MCI and HC (*p* <0.05), while CD14+/NLRP1+/PYCARD + and CD14+/NLRP1+/caspase1+ immune cells were augmented, although not significantly, in AD patients compared to other two groups. Finally, no differences could be observed in CD14+/NLRP1+/caspase 5+ cells (Fig. 4b). These results confirm those obtained in PCR analyses and indicate that, even if some inflammasome components are up regulated in MCI, fully functional inflammasomes are not assembled in this situation.

### Co-localization of inflammasome proteins in LPS-primed and Aβ_42−_stimulated monocytes by confocal microscopy analyses

**Fig. 5:**
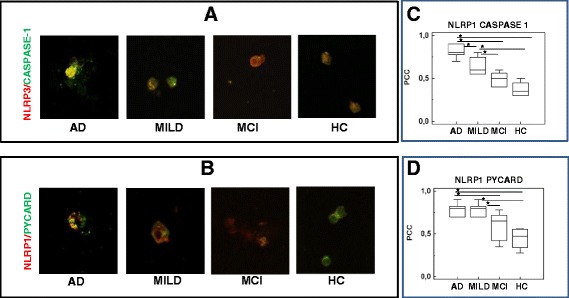
Confocal microscopy: co-localization of inflammasome proteins in CD14+ cells. Representative confocal fluorescence images (63× magnification) of experiments (N = 5) showing the effect of LPS-priming and Aβ_42_-stimulation on NLRP3/PYCARD (**a**) and NLRP1/caspase 1 (**b**) co-localization in monocytes of individuals with a diagnosis of either severe Alzheimer’s disease (AD), moderate Alzheimer’s disease (MILD) or Mild Cognitive Impairment (MCI) and of age- and sex-matched Healthy Controls (HC). Summary results of NLRP3/PYCARD and NLRP1/caspase-1 PCC (Pearson co-localization efficiency) are presented in (**c**) and (**d**), respectively. The boxes stretch from the 25 to the 75 percentile; the line across the boxes indicates the median values; the lines stretching from the boxes indicate extreme values. Statistical significance is shown *(*p* <0.05), **(*p* <0.01)

Aggregation and activation of inflammasome complexes was further analyzed by evaluating the co-localization of NLRP3 and NLRP1 with PYCARD, caspase 1 or caspase 8 by confocal microscopy. Co-localization efficiency was calculated using the Pearson co-localization coefficient (PCC). Results showed a clear co-localization of NLRP3 with PYCARD, caspase 1 and caspase 8 in LPS-primed and Aβ_42_-stimulated monocytes of AD patients alone; no co-localization could be detected in untreated cells (data not shown). In particular: 1) NLRP3/PYCARD, NLRP1/PYCARD and NLRP3/caspase1 co-localization was increased in both groups of AD patients compared to HC and MCI; 2), NLRP3/caspase8 and NLRP1/caspase5 co-localization was increased in severe AD alone; and 3) NLRP1/caspase1 co-localization was seen only in cells of MILD AD individuals. In all cases, with the exception of the NLRP1-caspase 1 complex, the highest PCC values were detected in cells of patients with severe AD disease (Fig. 5 and Table 1).

**Table 1:** Co-localization coefficient of inflammasome proteins. Pearson’s co-localization coefficient (PCC) of NLRP1 and NLRP3 with PYCARD, caspase-1, caspase-5 and caspase-8, in LPS-primed and Aβ_42−_stimulated CD14+ monocyte of patients with a diagnosis of either severe or moderate (MILD) Alzheimer’s Disease (AD) and mild cognitive impairment (MCI). Result obtained in and in age-and sex-matched healthy controls (HC) are also shown. Index reference values: PC range= −1 and +1

PCC	severe AD	MILD AD	MCI	HC
NLRP1+ PYCARD +	0.8^ac^ (0.7-0.82)	0.8 ^de^ (0.7-0.82)	0.5^ad^ (0.3-0.6)	0.4^ce (0.3–0.5)^
NLRP1+ CASPASE-1+	0.6 (0.4-0.7)	0.8 (0.7-0.84)	0.6 (0.4-0.65)	0.5 (0.5-0.7)
NLRP1+ CASPASE-5+	0.7^bac (0.5–0.8)^	0.4^b^ (0.23-0.5)	0.3^a^ (0.1-0.32)	0.3^c^ (0.2-0.5)
NLRP3+ PYCARD +	0.8^ac^ (0.6-0.82)	0.8 ^de^ (0.7-0.82)	0.6^ad^ (0.5-0.7)	0.5^ce^ (0.4-0.6)
NLRP3+ CASPASE-1+	0.9^bac^ (0.8-0.9)	0.8^bde^ (0.5-0.8)	0.5^ad^ (0.4-0.56)	0.3^ce^ (0.3-0.4)
NLRP3+ CASPASE-8+	0.8^ac^ (0.7-0.85)	0.6 (0.5-0.72)	0.5^a^ (0.3-0.55)	0.5^c^ (0.3-0.6)

Median values, interquartile range and statistical significances are presented (*p* <0.05)

^a^AD vs. MCI

^b^AD vs. MILD AD

^c^AD vs. HC

^d^MILD AD vs. MCI

^e^MILD AD vs. HC

### Inflammasome-related cytokines production in LPS-primed and Aβ_42−_-stimulated - monocytes of AD patients

**Fig. 6:**
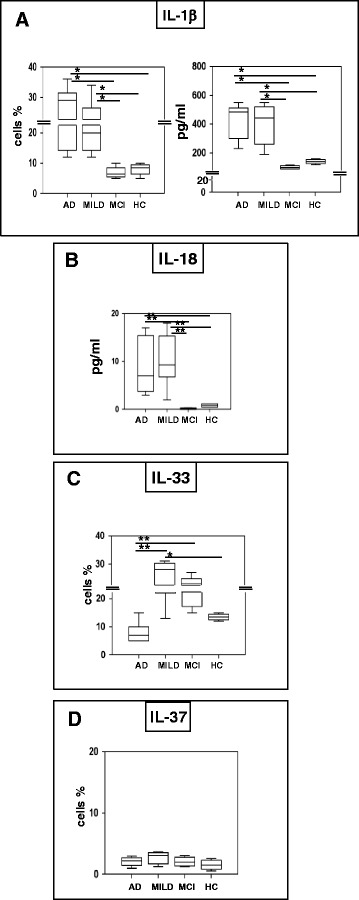
IL-1β, IL-18, IL-33 and IL-37 production. IL-1β, (panel **a**), IL-18 (panel **b**), IL-33 (panel **c**) and IL-37 (panel **d**). Interleukin-1β and IL-18 production was assessed by multiplex ELISA in supernatants. CD14+/IL-1β+, CD14+/IL-33 and CD14+/IL-37+ cells were analyzed by flow-cytometry. In both cases, LPS-primed and Aβ_42_-stimulated monocytes of individuals with a diagnosis of either severe Alzheimer’s disease (AD), moderate Alzheimer’s disease (MILD) or Mild Cognitive Impairment (MCI) and of age- and sex-matched Healthy Controls (HC) were analyzed. Summary results are shown in the bar graphs. The boxes stretch from the 25° to the 75°percentile; the line across the boxes indicates the median values; the lines stretching from the boxes indicate extreme values. Statistical significance is shown *(*p* <0.05), **(*p* <0.01)

Aggregation of the inflammasome results in the down-stream production of pro-inflammatory cytokines. Because FACS and confocal microscopy data indicated that the different subunits that compose the inflammasome do aggregate in LPS-primed and Aβ_42−_-stimulated- monocytes of AD patients, and PCR analyses showed that mRNA for these cytokines is increased in monocytes of AD patients, IL-1β, IL-18, IL-33 and IL-37 were next measured. Results showed that the concentration of IL-1β, IL-18 in supernatants was significantly increased in both groups of AD patients compared to MCI and HC (IL-1β *p* <0.05; IL-18 *p* <0.01)(Fig. 6a and b). CD14+/IL-1β+ cells were significantly increased as well in MILD and severe AD compared to MCI and HC (*p* <0.05)(Fig. 6a), whereas lack of proper reagents prevented IL18-expressing cells to be analyzed by FACS. The fact that IL-1β+ and IL-18 were not increased in MCI is not surprising considering that in this condition NLRPs but not PYCARD expression was augmented, thus preventing the assembly of a functional inflammasome; these data confirm the findings obtained with FACS and confocal analyses.

IL-33 could not be quantified in supernatants (data not shown), probably because the inflammatory mature form of this cytokines is not cleaved and secreted. Results obtained by flow-cytometry, nevertheless showed that CD14+/IL-33+ cells were increased in MCI and in MILD AD, with the lowest percentages of these cells seen in severe AD (*p* <0.01)(Fig. 6c). Notably this cytokine was shown to have a multifaceted protective role against AD and to negatively modulate NF-kB activity, dampening inflammation. Finally, CD14+/IL-37+ cells were marginally increased in AD compared to MCI and HC (Fig. 6d).

## Discussion

Inflammasomes are intracellular complexes formed by the assembly of multiple subunits that regulate the maturation and the secretion of pro-inflammatory cytokines [2]. At least four different inflammasomes are known; the NLRP3, in particular, is suspected to play a role in the pathogenesis of AD. Thus, in the APP/PSI animal model of AD, NLRP3 up regulation induces the production of IFN1β by microglia [14]; and, on the other hand, its deficiency results in a decreased deposition of amyloid-β [24, 26–29]. We have previously shown that peripheral monocytes of AD individuals are characterized by an inflammatory profile and a higher surface density of TLR molecules [30]. Further analyses performed in these same individuals allowed us to demonstrate that these cells also express binary complexes formed by Aβ peptides and MHC-molecules, possibly initiating Aβ-specific acquired immune reponses [31]. Herein we investigate the role of the inflammasome in the neuroinflammation that accompanies AD by analyzing peripheral immune cells of patients with MCI or AD. Results obtained using molecular, confocal and cytofluorimetic analyses indicated that the NLRP3 and NLRP1 inflammasomes are indeed activated in AD. The assembly of functional inflammasomes in AD was confirmed by the significantly increased amount of the proinflammatory cytokines IL-1β and IL-18 that were produced by LPS-primed and Aβ_42−_-stimulated- monocytes of AD patients.

Notably, NLRP1, NLRP3 and caspase 8, but not PYCARD were increased in individuals with a diagnosis of MCI, a condition that is often but not always prodromic to the development of AD. In this situation the assembly of a functional inflammasome is not possible. Consequently, neither a significant co-localization of inflammasome subunits nor increased amount of IL-1β and IL-18 were detected in these individuals. It is interesting to observe that in MCI a partial, initial up regulation of inflammasome proteins limited to NLRP proteins is observed. Only a percentage of MCI progresses to AD; these results thus allow the speculation that the inflammasome is somehow “primed” to be activated in all individuals with a diagnosis of MCI. An increased transcription of the other inflammasome components, the assembly of the functional complex, and the production of proinflammatory cytokines, occurs only in some cases, possibly because of still unknown “triggering” events that act on an “NLRP-primed” background.

NLRP3 and caspase 8 mRNA were up regulated in cells of severe AD patients; CD14+/NLRP3+/caspase8+ cells were increased as well in these individuals in whom the co-localization of caspase 8 with NLRP3 was also detected. Recent data showed that caspase 8 is not only an inducer of cell apoptosis but, together with the Fas-Associated protein with Death Domain (FADD), it interacts with NLRP3. Such interaction is required for caspase 1 activation, as well as for IL-1β and IL-18 secretion [5]. Confocal analyses confirmed that caspase 8 is present in the NLRP3 inflammasome complex, where it is involved in the cleavage of pro caspase 1 and IL-1β. These results support a direct role for caspase 8 in the processing of caspase 1 [6]. Our data confirm these findings, as well as recent results indicating that caspase 8 contributes to both NF-kB–dependent priming and post-translational activation of the NLRP3 inflammasome [5]. Increased NLRP1 and caspase 5 mRNA levels were also detected in cells of individuals with a diagnosis of severe AD. NLRP1 and caspase 5 colocalized in CD14 cells, and higher percentages of CD14+/NLRP1+/caspase 5+ immune cells were also seen in these patients. These data indicate that the NLRP1 inflammasome complex is also activated in severe AD; notably, recent results indicate that SNPs in the NLRP1 gene are associated with AD [32].

Inflammasome assembly leads to the production of proinflammatory cytokines; IL-1β and IL-18 mRNA and secretion were indeed significantly increased in AD. Interestingly, incubation of cells with Aβ_42_ was sufficient to induce IL-1β mRNA expression, but a significantly increased production of IL-1β and IL-18 was seen in LPS-primed and Aβ_42_-stimulated cells of AD patients alone. These findings indicate that such experimental conditions result in the assembly of fully functional inflammasomes only in cells of AD patients. Notably, transfection of human PBMC with siRNA specific for NLRP3 and PYCARD greatly reduced IL-1β production, confirming that the production of this cytokine is dependent on inflammasome assembly [33]. That IL-1β plays a role in the pathogenesis of AD has been repeatedly shown. To summarize: this cytokine induces a loss of phagocytic activity by the microglia [34], stimulates the hyperphosphorylation of tau protein [35] and affects synaptic plasticity. As a consequence of these effects, IL-1β can impair learning and memory processes [36, 37]. IL-18 has been involved in AD as well, even if fewer data are available. Thus, increased amounts of IL-18 were detected in the brain [38], plasma [39], and peripheral blood lymphocytes of AD [40], and the expression of the IL-18R complex is greatly augmented in peripheral blood cells of MCI and AD individuals [41]. Finally, recent data indicate that IL-18 directly stimulates Aβ production by human neuron-like cells, suggesting a key role for this cytokine in the pathogenesis of AD [42].

Two other novel cytokines, IL-33 and IL-37 were analyzed as well in this study; these cytokines are members of the IL-1β family and are produced upon activation of the inflammasome. Whereas no significant differences were seen in IL-37, mRNA levels of IL-33 were increased in both MILD AD and MCI compared to the values observed in severe AD. Analysis of peripheral CD14+/IL-33+ cells confirmed this observation, as these cells were increased both in MCI and MILD AD compared to severe AD and HC. IL-33 and IL-37 are dual function proteins with both intra and extra-cellular mechanisms of action as, besides being able to bind to their cognate receptors on target cells, they can act intracellularly as nuclear factor. IL-33 is reduced in AD brains [43]. This cytokine is believed to have a neuroprotective role secondary to the reduction of Aβ peptides secretion [44] and the activation of the phagocytosis of amyloid-β peptide by the microglia [45]. IL-33 also interacts intracellularly with the NF-kB p65 subunit [44]; the resulting IL-33/NF-kB p65 complex interferes with NF-kB–dependent transcription by impeding p65-mediated transactivation. This causes a negative modulation of NF-kB activity, with a dampening effect on inflammation [46]. The increase of IL-33 production in MCI and in MILD AD could thus be seen as an attempt of the immune response to reduce neuroinflammation. On the other hand, the observation that the levels of IL-33 seen in severe AD are comparable to those observed in elderly healthy individuals suggest that the IL33-dependent anti-inflammatory effects do not need to be activated in the absence of the pathological alterations present in AD.

Results herein stem from immunological analyses performed in peripheral blood immune cells; a number of data nevertheless provide evidence that the brain is invaded by peripherally derived monocytes/macrophages [47]. In murine models of Alzheimer’s disease, mice brains are infiltrated by bone marrow-derived macrophages that associate with amyloid plaques and clear Aβ deposits from the brain [48]. Our data indicate that the CD14+ cells that are likely present in the CNS of AD patients can carry active inflammasome complexes. It also has to be remembered that the role of inflammation in AD is ambiguous. Thus, whether this phenomenon is considered to be responsible for AD-associated neurodegeneration, an alternate hypothesis sees it as a monocyte/macrophages-mediated attempt to slow down the accumulation of Aβ plaques in the brain. Longitudinal studies and analyses performed in animal models will be needed to clarify this issue.

## Conclusions

NLRP3 up regulation in mice or human microglia cell line or in brain of AD patients has been previously reported [14, 24, 49], these are nevertheless the first data showing NLRP3 and NLRP1 inflammasome activation in Aβ stimulated peripheral monocytes of individuals with a diagnosis of AD. It will be interesting to verify whether these data could have a prognostic and/or diagnostic value in the clinical setting. In particular, the observation that some (NLRP1, NLRP3) but not not all (PYCARD, caspase 1) of the genes necessary for the assembly of an inflammosome complex genes are upregulated in MCI suggests that monitoring the transcription rate of, e.g. PYCARD in MCI could offer an early diagnostic tool for AD development. Migration of peripheral monocyte across the blood-brain barrier is likely an important factor in the neuroinflammation that accompanies Alzheimer’s disease. Very recent results showed that nucleoside reverse transcriptase inhibitors inhibit the activation of the inflammasome [50]. If neuroinflammation is deleterious in AD, these drugs could be an interesting tool in the treatment of this disease.

## Methods

### Patients and controls

Twenty individuals with a diagnosis of mild cognitive impairment (MCI) and a Mini- Mental State Examination (MMSE) score >24, 21 patients with moderate Alzheimer’s disease (MMSE score 19–23), 19 patients with severe Alzheimer’s disease (MMSE score <19) and 40 age-and sex-matched healthy controls (HC) were enrolled in the study. The clinical diagnosis of Alzheimer’s disease was performed according to the NINCDS-ADRDA work group criteria [51] and the DMS IV-R [52]. All Alzheimer’s disease patients underwent complete medical and neurological evaluation, as well as laboratory analysis, and CT scan or MRI. Additional investigations (e.g., EEG, SPECT scan, CSF examination, etc.) were performed in some cases to exclude reversible causes of dementia. Neuropsychological evaluation and psychometric assessment were performed with a neuropsychological battery that included the MMSE [53], Digit Span Forward and Backward, Logical Memory and Paired Associated Words Tests, Token Test, supra Span Corsi Block Tapping Test, Verbal Fluency Tasks, Raven Colored Matrices, the Rey Complex Figure, Clinical Dementia Rating Scale (CDR) [54], and the Hachinski Ischemic Scale. Individuals with a diagnosis of MCI were selected among subjects seen at our Memory Disorders Outpatients Service for the diagnostic evaluation of memory complaints without difficulties in daily activities. MCI diagnosis was based on Petersen’s criteria [55] as follows: 1) reported cognitive decline, 2) impaired cognitive function, 3) essentially normal functional activities, and 4) exclusion of dementia. The healthy controls were selected according to the SENIEUR protocol for immuno-gerontological studies of European Community’s Control Action Program on Aging [56, 57]. The cognitive status of HC was assessed by administration of MMSE (score for inclusion as normal control subjects >28). Written consent was obtained and ethical approval was granted by the Ethics Committee of the Don C Gnocchi Foundation in Milano, Italy.

### Blood sample collection and cell separation

Fifty ml of whole blood were collected in vacutainer tubes containing ethylenediaminetetraacetic acid (EDTA) (Becton Dickinson & Co., Rutherford, NJ, USA). Peripheral blood mononuclear cells (PBMC) were separated on lympholyte separation medium (Cedarlane, Hornby, Ontario, CA) and washed twice in PBS at 1500 RPM for 10 min; viable leukocytes were determined using a Scepter 2.0 Handheld Automated Cell Counter (Millipore, Billerica, MA).

### Cell cultures

PBMC (1 × 10^6^/ml) were cultured in 12 well cell culture plate (Greiner Bio-One GmbH Frickenhausen Germany) with RPMI 1640 supplemented with 10 % human serum, 2 mM L-glutamine, and 1 % penicillin (Invitrogen Ltd, Paisley, UK) for 2 h at 37 °C in a humidified 5 % CO_2_ atmosphere [58]. Non adhering PBMC were then washed away and monocytes were either resuspended in medium alone (medium), or were stimulated with: 1) 2 μg/ml Lipopolysaccharide (LPS) for 2 h (Sigma-Aldrich, St. Luis, MO, USA), 2) 10 μg/ml of 1–42 amyloid-beta peptide oligomer (Aβ_42_)(Sigma-Aldrich) for 24 h, or 3) primed with LPS (2 μg/ml) for 2 h before stimulation with 10 μg/ml of Aβ_42_ peptide oligomer for 24 h. In all cases cells were incubated at 37 °C in a humidified 5 % CO_2_ atmosphere. As it has been repeatedly shown, LPS pre-incubation is necessary to induce the intracellular accumulation of NLRP3 and pro-IL-1β, thus allowing the assembly of fully functional inflammasome complexes [13, 14, 59, 60]. For confocal microscopy analyses PBMC were cultured on chamber slide (Lab Tek Nalge Nunc Intern. Naperville IL USA) for 2 h at 37 °C in a humidified 5 % CO_2_ atmosphere [58]. Non adhering PBMC were then washed away and monocytes were either resuspended in medium alone or were stimulated with 1) 2 μg/ml LPS for 2 h (Sigma-Aldrich), 2) 10 μg/ml of Aβ_42_oligomer (Sigma-Aldrich) for 24 h, or 3) primed with LPS (2 μg/ml) for 2 h before stimulation with 10 μg/ml of Aβ_42_ peptide oligomer for 24 h. In all cases cells were incubated at 37 °C in a humidified 5 % CO_2_ atmosphere. Finally monocytes on chamber slide were fixed in 4 % paraformaldehyde in PBS for 15 min.

### RNA extraction and reverse transcription

RNA was extracted from unstimulated, LPS- or Aβ_42_- primed, or LPS-primed and Aβ_42_-stimulated-monocytes using the acid guanidium thiocyanate–phenol–chloroform method. RNA was dissolved in RNase-free water and purified from genomic DNA with RNase-free DNase (RQ1 DNase; Promega, Madison, WI). One microgram of RNA was reverse transcribed into first-strand cDNA in a 20 μl final volume containing 1 μM random hexanucleotide primers, 1 μM oligo dT and 200 U Moloney murine leukemia virus reverse transcriptase (Clontech, Palo Alto, CA). cDNA were evaluated for GAPDH expression by Real Time PCR to test RNA quality.

### Inflammasome signalling pathway

The human Inflammasomes RT^2^ Profiler PCR Array (Qiagen PAHS-097Z) was used to analyze gene expression of inflammasome components and signalling pathways. In this array a set of optimized primer assays allows the detection of mRNA transcripts of 84 genes as well as five housekeeping genes in a 96-well plate by real-time PCR. Individual specimens were pooled according to MMSE scores (severe AD, MILD AD, MCI, HC). cDNA from unstimulated cells and from cells stimulated with either LPS or Aβ_42_ alone or, finally, that had been LPS-primed and Aβ_42_ stimulated were grouped in different pools for PCR arrays. Data were analyzed by the comparative Ct method using GAPDH as the reference gene. Results are expressed as the fold changes between LPS or Aβ_42_ or LPS-primed and Aβ_42_ stimulated pools from each disease state and unstimulated condition. Heat maps were generated and genes hierarchically clustered by Euclidean distance and single linkage using TIGR MultiExperiment Viewer (MeV) v4.9 [61].

### Real time quantitative reverse transcription

Real Time quantitative Reverse Transcription PCR (RQPCR) was performed on a ABI Prism 7000 instrument (PE Applied Biosystems, Foster City, CA, USA) with gene specific primers and the SybrGreen chemistry to confirm the gene expression changes observed by arrays. All primers were cDNA specific and were purchased from Qiagen (Venlo, PB Venlo, The Netherlands). Specific PCR products amplification was detected using the RT2 SYBR Green Fluor with a 25 μl final volume of 12.5 μl RT2 qPCR Mastermix (Qiagen) 10.5 μl H2O, 1.0 μl of either diluted template or 1.0 μl RT2 qPCR Primer Assay. Results were expressed as ∆∆Ct and presented as ratios between the target gene and the GAPDH housekeeping mRNA. Experiments were run on each subject included in the study.

### Western blotting

Unstimulated and LPS-primed and Aβ_42_ stimulated-monocytes were washed and lysed with M-PER Mammalian Protein Extraction Reagent with phosphatase and protease inhibitors (10 μl/ml, Halt™ Protease and Phosphatase Inhibitor Cocktail, Thermo Scientific Inc, Rockford, IL, USA). After protein estimation using the Bradford Assay reagent (Bio-Rad) 20 μg of cell lysate was resolved by electrophoresis on a 10 % SDS-polyacrylamide gel under reducing conditions. Proteins were then transferred to PVDF membrane by electrophoretic blotting and blocked for 1 h in blocking buffer (Blocker BLOTTO Blocking Buffer, Thermo Scientific). Membranes were then incubated overnight with sheep anti-recombinant NALP3 (AF6789, R&D Systems) or for 1 h with sheep anti beta-actin antibody (AF4000, R&D Systems), then diluted 1:1000 in blocking buffer followed by HRP-conjugated anti-sheep IgG (HAF016, R&D Systems). Bound antibodies were visualized with a chemiluminescence development reagent (LiteAblot TURBO, Euroclone, Italy) according to the manufacturer’s instructions. Bands were imaged in a Gel-Doc Cabinet and the density of the image was measured. The density of each band was normalized with beta-actin (as housekeeping protein) and for each sample an arbitrary units (AU) value was calculated as follows: normalized density of NLRP3 band obtained from stimulated (LPS-primed+Aβ) monocytes/normalized density of NLRP3 band obtained from unstimulated monocytes.

### Flow cytometry immunofluorescent staining

Unstimulated and LPS-primed and Aβ_42_-stimulated-monocytes were stained with anti-CD14-PC7 (clone RMO52, isotype mouse IgG_2a_, Beckman Coulter, CA USA) mAb for 30 min at 4°. Cells were then washed, treated with FIX and PERM Cell kit (eBioscience, San Diego, CA, USA), and stained with anti- NLRP3-PE (clone 768319; isotype rat IgG_2a_ R&D Systems Inc. Minneapolis MN, USA), −NLRP1-APC (isotype rabbit polyclonal IgG Proteintech, Chicago, IL, USA), −caspase 5-FITC (isotype rabbit polyclonal; LifeSpan BioSciences,Inc, Seattle, WA USA), − PYCARD-FITC (clone HASC-71; isotype mouseIgG_1k_, eBioLegend San Diego CA,USA), IL-1β FITC(clone 8516; isotype mouse IgG_1_; R&D) IL-33-APC (clone 39412 isotype rat IgG_2B_ R&D), or IL-37-PE (clone 6A6, isotype mouse IgG_1k_ LSBioSciences, Inc) mAb for 30 min at 4°. The Fluorochrome Inhibitor of Caspases (FLICA) kit was used to analyze caspases. This kit uses a novel approach to detect active caspases. Once inside the cell, the FLICA inhibitor probe binds covalently to the active caspase (p20) alone. Staining of active caspase 1 and caspase 8 inhibitors was performed using the green fluorescent probe FAM-YVAD-FMK and FAM-LETD-FMK respectively, following the procedures suggested by the manufacturer (AM-FLICA, Immunochemistry, Bloomington, IN, USA). NLRP1, caspase 5 and PYCARD were conjugated using the Lightning-Link APC or FITC conjugation kit (Innova Biosciences, Cambridge, UK). Cells were finally analyzed using a Beckman-Coulter GALLIOS flow cytometer equipped with a single 15 mW argon ion laser operating at 488 nm and interfaced with CXP Software 2.1. 200,000 cells were acquired and gated on CD14 expression and side scatter properties. Isotype control or single fluorochrome-stained preparations were used for color compensation.

### Confocal microscopy analysis

Unstimulated, LPS- or Aβ_42_- primed, or LPS-primed and Aβ_42_-stimulated-PBMC were cultured on chamber slide (Lab Tek Nalge Nunc Intern. Naperville IL USA) for 24 h at 37 °C. Non-adherent cells were then washed away and monocytes were grown on chamber slides, fixed in 4 % paraformaldehyde in PBS for 15 min, and treated for 1 h at room temperature with FLICA staining of active caspase 1 or caspase 8 using green fluorescent FAM-YVAD-FMK probes, following the procedures suggested by the manufacturer (AM-FLICA). Cells were then treated with FIX and PERM Cell kit (eBioscience), and stained with PE or APC or FITC conjugated -mAbs specific for NLRP3 or NLRP1 and PYCARD or caspase 5 for 24 h at 4 °C. Finally cells were fixed with paraformaldehyde 1 % for 15 min, washed and mounted on slides using the Vectashield Mounting Medium (Vector Laboratories, Inc, Burlingame, CA, USA). Fluorescent images were acquired on a Leica TCS DMRE spectral laser-scanning confocal microscope (Leica Microsystems, Wetzlar, Germany) with the appropriate filters and laser (488, 633) and a 63× objective lens. Image analysis was performed using the Leica Confocal Software and co-localization index with ImageJ Software. Co-localization indexes were calculated using the plug in JACoP (Justo Another Co-localization Plugin) [62]. The summarized co-localization efficiency data was expressed as Pearson correlation coefficient (PCC) as previously described [63–65]. Briefly this coefficient measures the significance of true co-localization. The significance test evaluates the probability that the measured value of r obtained from the two colours is significantly greater than values of r that would be calculated if there were only random overlap. The test is performed by randomly scrambling the blocks of pixels (instead of individual pixels, because each pixel’s intensity is correlated with its neighbouring pixels) in one image, and then measuring the correlation of this image with the other (unscrambled) image (Costes et al.) The test produces values in the range −1 + 1, 0 indicating that there is no discernible correlation and −1 and +1 meaning strong negative and positive correlations, respectively.

### Cytokines in supernatants

Supernatants of unstimulated, LPS- or Aβ_42_- primed, or LPS-primed and Aβ_42_-stimulated-PBMC were analyzed. Interleukin-1β, IL-18 and IL-33 concentration were measured using the Human IL-1 β (Quantikine ELISA Kit DLB50; R&D Systems), Human IL-33 (Quantikine ELISA KitD3300; R&D Systems), and Human IL-18 (ELISA Kit -LS-F118-1; LifeSpan BioSciences, Inc.) according to the manufacturer’s recommendations.

### Statistical analyses

Quantitative data were not normally distributed (Shapiro–Wilk test) and are thus summarized as median and Interquartile Range (IQR) (25° and 75° percentile). Comparisons between groups were analyzed used a Kruskal-Wallis ANOVA for each variable. Comparisons among the different groups were made using a 2-tailed Mann-Whitney *U* test performed for independent samples. Western Blot data were normally distributed, and were summarized as mean ± standard error. In this case, comparisons were performed using ANOVA and unpaired Student’s *t* test. Data analysis was performed using the MedCalc statistical package (MedCalc Software bvba, Mariakerke, Belgium).

## Abbreviations

IL: interleukin
Aβ: amyloid-β
MCI: mild cognitive impairment
AD: Alzheimer’s disease
HC: healthy controls
NLRs: Nod-like receptors
PAMPs: pathogen-associated molecular patterns
DAMPs: damage-associated molecular pattern molecules
ROS: reactive oxygen species
FADD: Fas-Associated protein with death domain
LTP: long-term potentiation
MMSE: mini- mental state examination
PBMC: peripheral blood mononuclear cell
FACS: fluorescence-activated cell sorting
